# Biopsy series of acute kidney injury from a tertiary care referral center in south India

**Published:** 2015-01-01

**Authors:** Siddappa Sujatha, Kowalya Ramprasad

**Affiliations:** ^1^Department of Pathology, Institute of Nephrourology, Victoria Hospital Campus, Bangalore, Karnataka, India; ^2^Department of Biochemistry, Institute of Nephrourology, Victoria Hospital Campus, Bangalore, Karnataka, India

**Keywords:** Acute kidney injury, Kidney biopsy, Nephritis

## Abstract

Acute kidney injury (AKI) is common in hospital patients and more so in critically ill patients. It is frequent, harmful and potentially treatable condition. In a total of 243 renal biopsies 130 cases fulfilled the criteria of acute kidney injury. The usual mode of presentation was renal failure followed by acute nephritis. Histopathologically acute interstitial nephritis was the usual finding followed by post infectious-glomerular nephritis. The acute renal failure (ARF) prognosis is influenced by the co-morbidity states and we had a high mortality of 8.46% in our referral centre.

**Implication for health policy/practice/research/medical education:**

Acute kidney injury (AKI) is common in hospital patients and very common in critically ill patients. It is common, harmful and potentially treatable condition. In a total of 243 renal biopsies 130 cases fulfilled the criteria of acute kidney injury. The most common mode of presentation was renal failure followed by acute nephritis. Histopathologically acute interstitial nephritis was the most common finding followed by post infectious-glomerular nephritis. The acute renal failure prognosis is influenced by the co-morbidity states and we had a high mortality of 8.46% in our referral center.

## Introduction

Acute kidney injury (AKI) is a broad clinical syndrome with important clinical consequences including death. It is common, harmful and potentially treatable condition. The definition for AKI is evolving and is defined as an abrupt decrease in kidney function that includes, but not limited to acute renal failure (1). Numerous preventable risk factors lead to AKI but irrespective of its nature, AKI is a predictor of immediate and long-term adverse outcomes, and hence early detection and potential prevention is of paramount importance (2,3).

An increasing prevalence of acute (and chronic) kidney disease worldwide and more so in developing countries with limited resources warrants addressing the detection of AKI in its early and potentially reversible stages to prevent the disease progressing to kidney failure necessitating renal replacement therapy. It is also not very clear whether AKI is common setting in critical illness and ICU its mortality rate high (4).

Here we present a cliniopathological review of cases with AKI from an exclusive nephro-urology referral tertiary care institute from South India.

## Materials and Methods

The Institute of NephroUrology is a premier organization promoting health care services exclusively in the field of nephrology and urology. It is a non-profit tertiary care referral institute funded by government of Karnataka, India.

Our hospital mainly caters to the need of the rural population and the population below the poverty line. Our 2012 annual nephrology outpatient has been 19762 cases and inpatients admissions are 1976 cases of which 80% have been patients below the poverty line with funding from the government. Most of the cases to our hospital are referral cases and acute cases. Referred cases are followed up at their primary center as there is no facility for maintenance dialysis and suitable patients are advised transplant programmes.

We under took this retrospective analysis of all the patients who underwent percutaneous renal biopsies in our tertiary care referral center over the past year. A total of 243 renal biopsies from January 2012 to March 2013 at our center were included in this study. Base line demographics, clinical history along with histopathological data were analyzed.

AKI was diagnosed by oliguria, azotemia (serum creatinine >1.5 mg/dl or 0.3 mg/dl increase above the baseline) with or without the requirement of dialysis. Renal biopsy was done if patient was oliguric or dialysis dependent at the end of 3 weeks or post mortem.

### Data analysis

Patient demographics, clinical information and laboratory data have been collected from the patient clinical records. The results were tabulated on the computer and data has been presented as mean and standard deviation.

### Ethical issues

The study was approved by the institutional ethics committee of our University. A written informed consent was obtained from all the study participants.

## Results

### Statistical analysis

Out of 243 biopsies, 130 cases fulfilled the criteria of acute kidney injury. The mean age was 38.8 ±10.02 years with male preponderance (73 males to 57 females). The most common mode of presentation was renal failure (serum creatinine >1.4 mg/dl in 54.6%) followed by acute nephritis (22.3%). Histopathologically acute interstitial nephritis was the most common finding in 40.8% patients followed by post infectious glomerular nephritis (19.2%) and ischemia causes (6.9%).

### Results of acute on chronic renal failure patients

The risk of acute renal injury in chronic renal failure cases who have been biopsied are as follows: We had a 15 male to 13 female patients cases respectively, with nine cases of nephrotic syndrome, eight cases of hypertension, four cases of diabetes mellitus, three cases of congestive cardiac failure, two cases of sepsis and one case of chronic liver disease. Of these chronic kidney disease (CKD) patients 28 cases had documented proteinuria.

### Results of cases AKI related to pregnancy

We had 4 cases of AKI related to pregnancy which required biopsy. Of these cases, one case had associated anti-GBM disease; the other three cases were of sepsis. All these patients did not have any prior renal insufficiency of any cause, history of hypertension or diabetes in the past, history of renal stone disease in the past, renal scarring on ultrasonography, small sized kidney, single kidney, elevated creatinine.

### Results in the critically ill in the ICU

At our institute 70 patients of urology and 433 patients with nephrology complaints presented with acute renal failure requiring intensive care treatment. Of these patients 145 nephrology patients and 18 urology patients succumbed to the illness while 208 patients required dialysis.

Among the 208 cases 160 patients were weaned of dialysis whereas the rest of the patients were dialysis dependent for more than three months and were later lost for follow up. The remaining 80 nephrology and 17 urology patients were discharged against medical advice, 35 urology patients required 2-3 cycles of dialysis and were later stabilized and discharged. The rest of 56 cases were mostly of obstructive uropathy complications and did not require dialysis.

## Discussion

In contrast to developed countries where most cases of AKI are related to trauma and multi-organ failure, AKI in developing countries is often related to medical causes (5). An increasing prevalence of acute and CKD worldwide and more so in developing countries warrants addressing the detection of AKI in its early and potentially reversible stages to prevent the disease progression to kidney failure necessitating renal replacement therapy (6). These cases of AKI are amongst the most potent predictors of acute decline of renal function following co-morbid condition known to be associated with high mortality and morbidity (7). Its also not very clear why AKI in the common setting of critical illness and ICU has a high mortality rate. Though documented as an important clinical diagnosis amongst hospitalized patients, there is an increase in cases of AKI worldwide and problems of kidney disease are on rise universally (8,9).

In our study AKI was very common in critically ill patients. Many of the cases were of the more acute and reversible nature associated AKI. In these patients, AKI was most often secondary to extra renal events such as congestive cardiac failure, bundle branch block, chronic respiratory airway disease, sepsis, metabolic disturbances while most of the urological cases were due to obstructive uropathy. In five patients the AKI was drug related while in four females AKI was due to puerperal sepsis. In our study acute interstitial nephritis was the most common cause in 40.8% patients followed by postglomerular nephritis (19.2%) and ischemia causes (6.9%).

AKI is associated with poor patient outcomes. In our study 8.46% of the patients succumbed to the complications of kidney injury. The majority of cases of AKI cannot be anticipated, until we can effectively identify truly high-risk subjects, using a combination of clinical risk factors and one or more biomarkers. There is increased recognition that AKI is encountered in multiple settings and in all age groups, and that its course and outcomes are influenced by the severity and duration of the event (10,11). Evidence suggests that patients who have had AKI are at increased risk of subsequent CKD (12). These emerging data point to an urgent need for a global effort to highlight that AKI is preventable, its course is modifiable, and its treatment can improve outcomes.

## Conclusion

We found a high mortality of 8.46% in our referral centre. Is renal support underutilized or delayed? This is the question of the hour.

## Acknowledgements

Authors would like to thank Dr. Mytri KM for processing the cultures of some of the patients listed in our biopsy series. We thank Dr. CS Ratkal Director Institute of NephroUrology and all our clinical colleagues.

## Conflict of interests

The authors declared no competing interests.

## Ethical considerations

Ethical issues (including plagiarism, data fabrication, double publication) have been completely observed by the authors.

## Funding/Support

None.

## Authors’ contributions

SS is the reporting pathologist for all the biopsies and KR has tabulated data, helped in literature review retrieval and reference search.
